# Auto-Ubiquitination-Induced Degradation of MALT1-API2 Prevents BCL10 Destabilization in t(11;18)(q21;q21)-Positive MALT Lymphoma

**DOI:** 10.1371/journal.pone.0004822

**Published:** 2009-03-12

**Authors:** Heidi Noels, Riet Somers, Hongxiang Liu, Hongtao Ye, Ming-Qing Du, Christiane De Wolf-Peeters, Peter Marynen, Mathijs Baens

**Affiliations:** 1 Human Genome Laboratory, Department for Molecular and Developmental Genetics, VIB, Leuven, Belgium; 2 Human Genome Laboratory, Center for Human Genetics, Molecular Genetics, University of Leuven, Leuven, Belgium; 3 Division of Molecular Histopathology, Department of Pathology, University of Cambridge, Cambridge, United Kingdom; 4 Department of Morphology and Molecular Pathology, University of Leuven, Leuven, Belgium; East Carolina University, United States of America

## Abstract

**Background:**

The translocation t(11;18)(q21;q21) is the most frequent chromosomal aberration associated with MALT lymphoma and results in constitutive NF-κB activity via the expression of an API2-MALT1 fusion protein. The properties of the reciprocal MALT1-API2 were never investigated as it was reported to be rarely transcribed.

**Principal Findings:**

Our data indicate the presence of *MALT1-API2* transcripts in the majority of t(11;18)(q21;q21)-positive MALT lymphomas. Based on the breakpoints in the *MALT1* and *API2* gene, the MALT1-API2 protein contains the death domain and one or both immunoglobulin-like domains of MALT1 (∼90% of cases) - mediating the possible interaction with BCL10 - fused to the RING domain of API2. Here we show that this RING domain enables MALT1-API2 to function as an E3 ubiquitin ligase for BCL10, inducing its ubiquitination and proteasomal degradation *in vitro*. Expression of *MALT1-API2* transcripts in t(11;18)(q21;q21)-positive MALT lymphomas was however not associated with a reduction of BCL10 protein levels.

**Conclusion:**

As we observed MALT1-API2 to be an efficient target of its own E3 ubiquitin ligase activity, our data suggest that this inherent instability of MALT1-API2 prevents its accumulation and renders a potential effect on MALT lymphoma development via destabilization of BCL10 unlikely.

## Introduction

**Figure 1:**
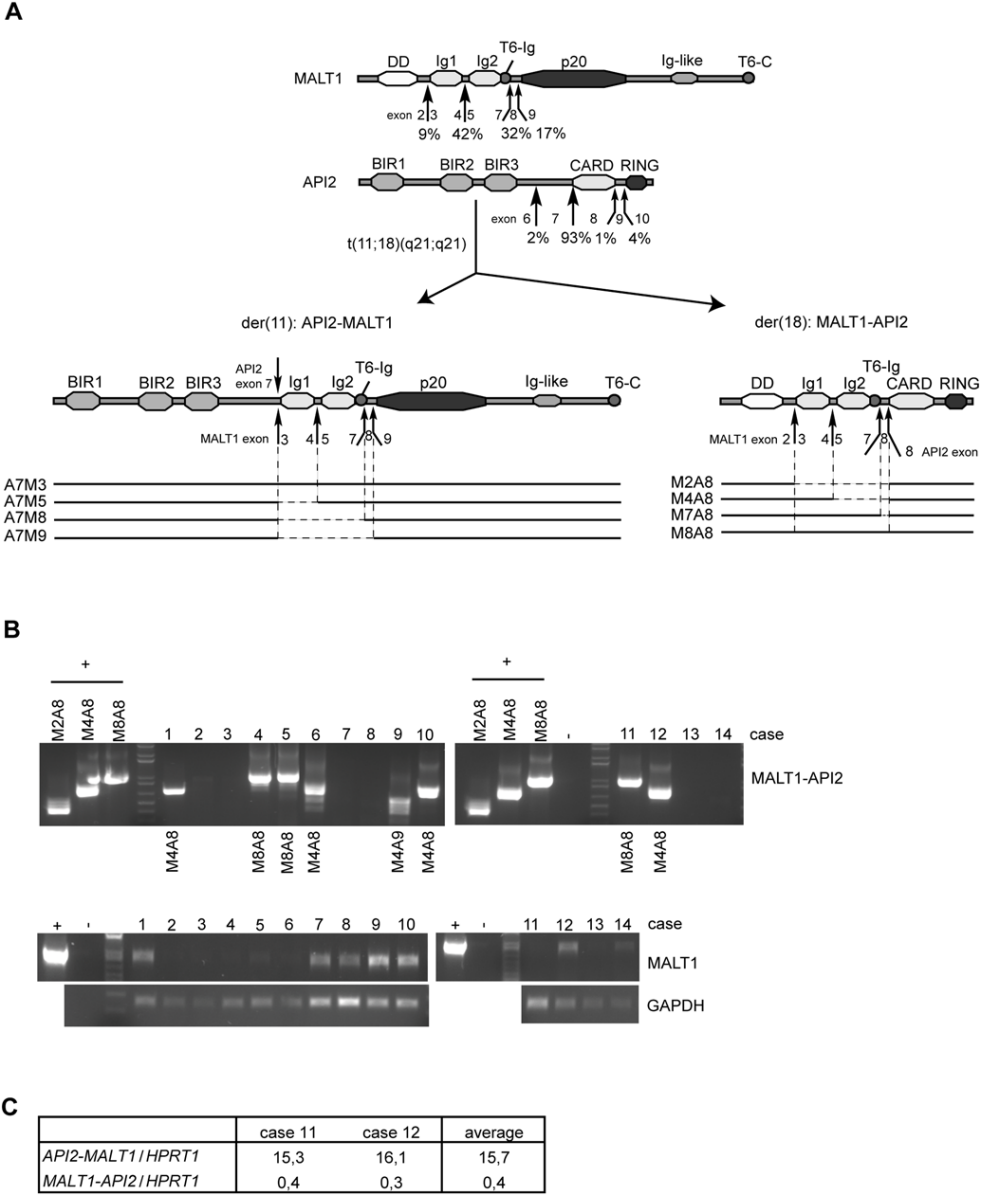
RT-PCR analysis of *MALT1-API2* expression in t(11;18)(q21;q21)-positive MALT lymphoma. A, Features of MALT1, API2, and the fusion proteins API2-MALT1 and MALT1-API2. Indicated is the domain content (solid bars) of the API2-MALT1 fusion variants A7M3, A7M5, A7M8 and A7M9 (fusion *API2* exon 7 to *MALT1* exon 3/5/8/9, respectively) and their corresponding MALT1-API2 fusion variants M2A8, M4A8, M7A8 and M8A8 (fusion *MALT1* exon 2/4/7/8, respectively, to *API2* exon 8). B, Shown are the amplification products of nested PCR reactions on cDNA extracted from 14 t(11;18)(q21;q21)-positive MALT lymphoma cases (see Table 1). A PCR on pcD-F-M2A8, pcD-F-M4A8 and/or pcD-F-M8A8 was performed as positive control, as indicated. An RT-PCR for *MALT1* and *GAPDH* was included for RNA quality control. C, Results of quantitative RT-PCR for cases 11 and 12 for *MALT1-API2*, *API2-MALT1* and *HPRT1*. For primers used, see Materials and Methods and Table 2.

Extranodal marginal zone B-cell lymphoma of mucosa-associated lymphoid tissue (MALT), or MALT lymphoma, represents 7–8% of all B-cell lymphomas. Two translocations specifically associated with MALT lymphoma are t(1;14)(p22;q32) and t(14;18)(q32;q21), which upregulate the expression of the *BCL10* [1], [2] and the *MALT1* [3], [4] gene, respectively, via their fusion to the *IgH* enhancer. The most important translocation, present in ∼40% of pulmonary and ∼10–30% of gastric MALT lymphomas, is t(11;18)(q21;q21), which rearranges the *API2* and the *MALT1* gene [5], [6]. In approximately half of the cases, the resulting API2-MALT1 fusion protein contains the three N-terminal baculovirus IAP repeat (BIR) domains of API2 fused to the C-terminal caspase-like domain of MALT1. Depending on the breakpoint in the *MALT1* gene however, the fusion protein can contain in addition one or two immunoglobulin (Ig)-like domains of MALT1, as observed in ∼40% and ∼10% of the cases, respectively (Fig 1A) [7].

API2 belongs to the inhibitor of apoptosis protein (IAP) family and has been implicated in anti-apoptotic processes, with an important role for its BIR domains as ‘protein sink’ for apoptosis mediators like caspase-3 and -7 [8]. In addition, a C-terminal really interesting new gene (RING) domain confers API2 with an E3 ubiquitin ligase activity [9], and ubiquitination-induced degradation of pro-apoptotic proteins like SMAC/DIABLO [10] might contribute significantly to its anti-apoptotic function.

MALT1 [11], [12] and BCL10 [13] on the other hand are important players in the signalling cascade from the antigen receptors on B- and T-cells to the transcription factor NF-κB. Upon T-cell receptor (TCR) triggering, CARMA1 recruits the BCL10-MALT1-TRAF6 complex to the lipid rafts surrounding the receptor [14]–[17]. Raft-mediated oligomerization activates the E3 ubiquitin ligase activity of TRAF6, resulting in Lys63-linked polyubiquitination of MALT1 [18] and BCL10 [19]. This mediates the recruitment of the IKKα/IKKβ/IKKγ complex to the CARMA1-BCL10-MALT1 complex [18], [19] and induces full NF-κB activation via the phosphorylation of IKKβ, together with the Lys63-linked polyubiquitination of IKKγ [20]. The important function of MALT1 and BCL10 in antigen receptor signalling to NF-κB suggests a role for enhanced NF-κB activation in MALT lymphoma development.

Also API2-MALT1 has been linked to constitutive NF-κB activation [21], [22] via polyubiquitination of IKKγ [23], [24]. The permanent association of the fusion protein with the lipid rafts [24], [25] or its oligomerization [23] via the first BIR domain bypasses the normal requirement of BCL10-mediated oligomerization of the MALT1-TRAF6 complex, resulting in TRAF6-mediated NF-κB activation without the need for antigen receptor triggering [26]. NF-κB activation by API2-MALT1 has been shown to upregulate anti-apoptotic genes [25], [27]. In addition, API2-MALT1 retains the ability of API2 to interact with SMAC/DIABLO [28], suggesting that API2-MALT1 also interferes indirectly with apoptotic signalling. In contrast to API2 however, API2-MALT1 has no E3 ubiquitin ligase activity and cannot ubiquitinate and target BCL10 for proteasomal degradation [29]. This could explain the increased BCL10 expression levels in MALT lymphomas with t(11;18)(q21;q21) [29], which, together with the unusual nuclear localization of BCL10 in these lymphomas [30], [31] have been suggested to contribute to the oncogenic character of this translocation. Altogether, these data point to divergent roles for API2-MALT1 in MALT lymphomagenesis.

In contrast to API2-MALT1, the reciprocal fusion *MALT1-API2* produced on the der(18) of the t(11;18)(q21;q21) escaped attention as initial reports found *MALT1-API2* RNA not to be consistently expressed [32]–[34]. Analyses of the genomic breakpoints showed that although the translocation is accompanied by deletions, duplications and insertions, a MALT1-API2 fusion protein can be formed in the majority of cases [35], [36]. This raised the question whether the reciprocal fusion protein might hinder MALT lymphomagenesis and induce its own transcriptional silencing.

MALT1-API2 always contains the N-terminal death domain (DD) of MALT1 fused to the C-terminal RING domain of API2. Variability of the breakpoints in *MALT1* generates MALT1-API2 fusion variants that can contain in addition one or two Ig-like domains of MALT1 (Fig 1A) [35], [36]. We recently showed that the DD and Ig-like domains of MALT1 cooperate for BCL10 binding, whereas two functional TRAF6 binding sites in MALT1, the first one directly 3′ of the second Ig-like domain (T6-Ig) and the second in the MALT1 C-terminus (T6-C), are involved in TRAF6 recruitment [26]. The presence of these BCL10 and TRAF6 binding sites in some of the MALT1-API2 fusion variants in combination with the RING E3 ubiquitin ligase domain of API2 suggests that these MALT1-API2 proteins could bind BCL10 and/or TRAF6 and mark them for ubiquitination-induced degradation.

In this study we provide evidence that *MALT1-API2* expression is not generally silenced in t(11;18)(q21;q21)-positive MALT lymphomas. We furthermore show that the MALT1-API2 fusion variants with one or both Ig-like domains of MALT1 do bind BCL10 though fail to interact with TRAF6. The RING domain in addition provides MALT1-API2 with an E3 ubiquitin ligase activity, allowing the MALT1-API2 variant with one Ig-like domain to trigger ubiquitination and proteasome-mediated degradation of BCL10 *in vitro*. However, the RING domain also destabilizes MALT1-API2 itself via auto-ubiquitination-induced degradation. This inherent instability of MALT1-API2 most likely limits its protein level in t(11;18)(q21;q21)-positive MALT lymphomas and thus renders a possible effect on their development via destabilization of BCL10 unlikely.

## Results

### *MALT1-API2* expression in *MALT* lymphoma with t(11;18)(q21;q21)

Although initial reports found *MALT1-API2* not to be expressed in the t(11;18)(q21;q21)-positive MALT lymphoma cases analyzed, no correlation was made with the genomic architecture at the breakpoint on the der(18) chromosome in these cases [32]–[34]. We therefore analyzed via RT-PCR expression of *MALT1-API2* in 14 cases of t(11;18)(q21;q21)-positive MALT lymphoma, of which the fusion junctions on der(18) had previously been characterized at the genomic level in 10 of these cases (Table 1) [35], [36]. *MALT1-API2* transcripts were identified in 8 out of the 14 cases (case 1–14, Fig 1B, Fig S1AB, Table 1). Sequencing of the PCR products was performed to identify the *MALT1-API2* fusion variant and confirmed the generation of in-frame fusions in all cases. In the 6 cases for which no *MALT1-API2* fusion could be detected via RT-PCR, one case (case 7) did not reveal a *MALT1-API2* fusion at the genomic level either [36], while for 4 other cases (case 2, 3, 13 and 14), the absence of *MALT1-API2* transcripts most likely reflects poor RNA quality as also no *MALT1* transcript could be amplified (Fig 1B). In total, for 7 of the 9 cases for which previously a *MALT1-API2* fusion was demonstrated at the genomic level, the *MALT1-API2* variant could be confirmed at the transcript level (Table 1).

**Table 1:** Characteristics of the MALT lymphoma cases examined.

case no.	previous no.	site of tumour	*API2-MALT1* variant at transcript level(3)	*MALT1-API2* variant at genomic level(3)	RT-PCR+sequencing	BCL10 nuclear expression
1	case 2(1)	stomach	A7M5	M4A8	M4A8	+
2		stomach	A7M8	na	−	+
3	case 3(1)	stomach	A7M5	M4A8	−	na
4		stomach	A7M9	na	M8A8	+
5	case 18(1)	small intestine	A7M9	M8A8	M8A8	+
6	case 8(1)	lung	A7M5	M4A8	M4A8	+
7	case 16(1)	stomach	A7M8	−	−	+
8	case 17(1)	stomach	A7M8	M4A8	−	+
9	case 10(1)	stomach	A7M5	M4A9	M4A9	+
10	case 5(1)	stomach	A7M5	M4A8	M4A8	+
11	case 5(2)	stomach	A7M9	M8A8	M8A8	na
12	case 3(2)	stomach	A7M5	M4A8	M4A8	na
13		stomach	A7M8	na	−	na
14		spleen	A7M5	na	−	na
*15*		stomach	*A7M8*	*na*	−	*na*

(1) case number in [36] *data not shown for case 15*.

(2) case number in [35].

(3) data published in [35], [36].

na = not analyzed.

− = not found.

Quantitative RT-PCR furthermore showed that although *MALT1-API2* is much lower expressed than *API2-MALT1*, its expression level is still about half that of *HPRT1* and thus of potential biological significance (Fig 1C). The high expression level of *API2-MALT1* reflects its potential to upregulate its own expression via activation of NF-κB, which drives transcription from the *API2* promoter [37].

In conclusion, these data argue against silencing of *MALT1-API2* transcription in t(11;18)(q21;q21)-positive MALT lymphomas.

### BCL10 but not *TRAF6* interacts with Ig-like domain-containing *MALT1-API2* variants

**Figure 2:**
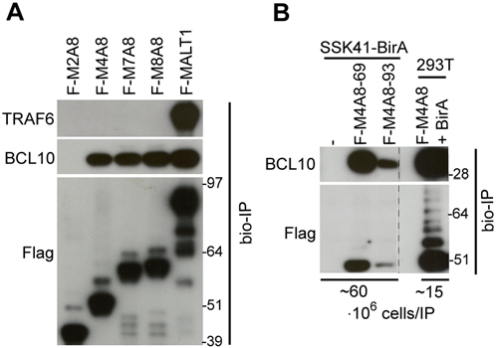
MALT1-API2 interacts with BCL10 via the MALT1 Ig-like domains. A, Inclusion of one or two Ig-like domains of MALT1 in MALT1-API2 allows the binding of endogenous BCL10 in HEK-293T. Immunoblot with α-TRAF6, α-BCL10 and α-Flag on protein complexes co-precipitated with Flag-bio-tagged M2A8, M4A8, M7A8, M8A8 and MALT1, transiently expressed in HEK-293T cells. B, M4A8 binds endogenous BCL10 in SSK41 B-cells. Immunoblot with α-BCL10 and α-Flag on protein complexes pulled down with Flag-bio-tagged M4A8 stably expressed in SSK41-BirA B-cells. Precipitated protein complexes from two different SSK41-BirA-M4A8 clones (69 and 93) were compared to the protein complex from HEK-293T cells with transient overexpression of M4A8. Molecular weight standards are in kDa.

MALT1 requires its DD and Ig-like domains to bind BCL10, while TRAF6 is recruited to TRAF6 binding sites in the second Ig-like domain (T6-Ig) and the C-terminus (T6-C) of MALT1 (Fig 1A) [26]. To examine whether MALT1-API2 also recruits BCL10 and/or TRAF6, we transiently expressed the different MALT1-API2 fusion variants fused to a biotinylation (bio) tag together with the *E. coli* BirA biotin protein ligase in HEK-293T cells. Protein complexes of biotinylated MALT1-API2 were subsequently pulled-down with streptavidin-coated paramagnetic beads, as recently described [38]. While M2A8 (Fig 1A, fusion of exon 2 of *MALT1* to exon 8 of *API2*) did not bind BCL10, the inclusion of one or two Ig-like domains of MALT1 in the MALT1-API2 fusion variants M4A8 and M7A8/M8A8, respectively (Fig 1A, fusion of exon 4, 7 and 8 of *MALT1*, respectively, to exon 8 of *API2*) resulted in co-precipitation of endogenous BCL10, similarly as for MALT1 (Fig 2A). In contrast, the presence of the TRAF6 binding site T6-Ig of MALT1 in M7A8 and M8A8 did not allow the recruitment of endogenous TRAF6, while TRAF6 efficiently co-precipitated with MALT1 (Fig 2A). To exclude cell-type specific interactions, we generated SSK41 Marginal Zone lymphoma B-cells stably expressing the BirA biotin ligase together with the bio-tagged M4A8 fusion variant. Streptavidin-mediated pull-down again showed an efficient co-purification of endogenous BCL10 with M4A8, despite its lower expression level in SSK41 compared to HEK-293T cells (Fig 2B).

In conclusion, our data show that the combination of the DD with one or two Ig-like domains of MALT1 in MALT1-API2 facilitates the interaction with BCL10, but not with TRAF6.

### *M4A8* ubiquitinates BCL10 via its RING domain

**Figure 3:**
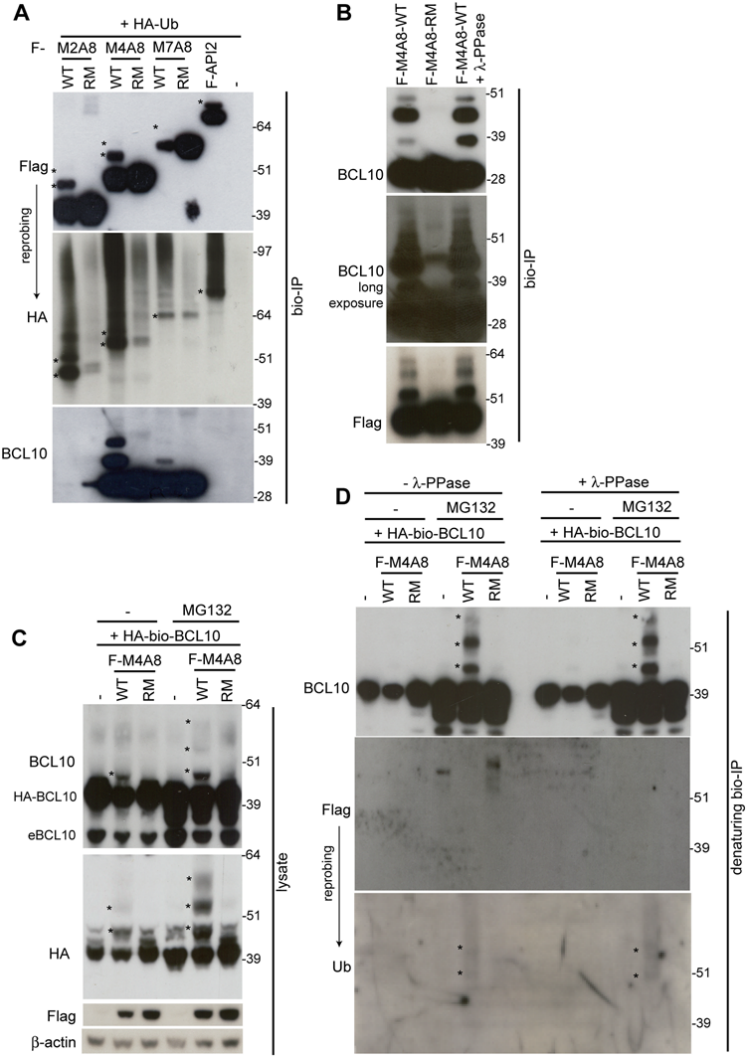
BCL10 is a target of the RING E3 ubiquitin ligase activity of MALT1-API2 variants with MALT1 Ig-like domains. A–B, Flag-bio-tagged M2A8, M4A8 and M7A8 (wild-type (WT) or RING mutant (RM)), API2 and empty vector were transiently expressed together with HA-Ub in HEK-293T. Protein complexes were precipitated with streptavidin-coated paramagnetic beads and analyzed with α-Flag, α-HA (after stripping of α-Flag) and α-BCL10. A, Mono- and di-ubiquitinated bands of WT MALT1-API2 and API2 are indicated with asterisks in the α-Flag and α-HA panel. B, Where indicated, the precipitated protein complex was treated with λ-PPase to remove phosphorylation. C–D, BCL10 ubiquitination by M4A8 becomes more pronounced after inhibition of the proteasome. HA-bio-tagged BCL10 was transiently overexpressed in HEK-293T together with Flag-tagged M4A8 (WT or RM) or empty vector. Where indicated, cells were pretreated with MG132 to inhibit to proteasome. C, Cell lysates were immunodetected with α-BCL10 and α-HA (both for HA-BCL10), α-Flag and α-β-actin. BCL10 ubiquitination is indicated with asterisks. D, Following denaturation of the cell lysates, HA-bio-tagged BCL10 was precipitated via streptavidin pull-down and where indicated, treated with λ-PPase to remove phosphorylation. The bio-IPs were then immunodetected with α-BCL10, α-Flag (to check if the denaturation was complete) and α-Ub. BCL10 ubiquitination is indicated with asterisks. Molecular weight standards are in kDa.

The RING domain of API2 enables it to function as an E3 ubiquitin ligase, capable of auto-ubiquitination [9], [39] and ubiquitination of interacting proteins [10], [40]–[42], like BCL10 via its interaction with the first BIR domain of API2 [29]. We thus wondered whether also MALT1-API2 possessed E3 ubiquitin ligase activity. The different bio-tagged MALT1-API2 fusion variants were therefore transiently expressed in HEK-293T cells together with HA-tagged ubiquitin (Ub) and subsequently purified via streptavidin-mediated pull-down. Western blot analysis with α-Flag (for API2 and MALT-API2) and α-HA (for HA-Ub) clearly showed that all MALT1-API2 fusion variants were ubiquitinated, similarly as API2 (Fig 3A). Furthermore, mutation of the critical His and the two flanking Cys residues of the RING domain (RING mutant; RM) abolished most ubiquitination (Fig 3A), indicating auto-ubiquitination of MALT1-API2. Interestingly, cellular BCL10 co-purified with bio-tagged M4A8, and to a much lesser extent with M7A8, showed a similar pattern of ubiquitination, which again was completely dependent on a functional RING domain in MALT1-API2 (Fig 3A). Remarkably, we were not able to co-precipitate BCL10 together with bio-tagged full-length API2 (Fig 3A), in contrast to a recent report on the BIR-mediated interaction of BCL10 with API2 [29]. To exclude that the slower migrating BCL10 bands observed in Fig 3A resulted from BCL10 phosphorylation, protein complexes co-purified with bio-tagged wild-type (WT) M4A8 were treated with λ-PPase, but this did not affect the migration pattern of BCL10 (Fig 3B).

To further investigate these BCL10 modifications by M4A8, we transiently expressed HA-bio-tagged BCL10 in HEK-293T cells, alone or in combination with Flag-tagged WT- or RM-M4A8. While no effect of the RING mutant M4A8 on BCL10 was seen in the cell lysate, co-expression of BCL10 with wild-type M4A8 again induced BCL10 modifications (indicated with asterisks, α-BCL10 and α-HA panel Fig 3C). When the cells were treated with the proteasomal inhibitor MG132 prior to harvesting, the BCL10 modifications became more pronounced, further suggesting that M4A8 induces BCL10 ubiquitination (Fig 3C). This was even more obvious when BCL10 was purified via streptavidin pull-down in denaturing conditions to destroy existing protein interactions (α-BCL10 panel Fig 3D, left part). Again, phosphorylation could be excluded, since treatment of the purified BCL10 proteins with λ-PPase did not affect the slower migrating BCL10 bands (Fig 3D, right part). In contrast, these bands could be revealed with an α-Ub antibody (Fig 3D).

Altogether, these data indicate that MALT1-API2 can function as an E3 ubiquitin ligase via the RING domain of API2. While all fusion variants auto-ubiquitinate, only M4A8 is able to induce significant BCL10 ubiquitination in overexpression experiments.

### Ubiquitination of BCL10 by *M4A8* induces its degradation by the proteasome

**Figure 4:**
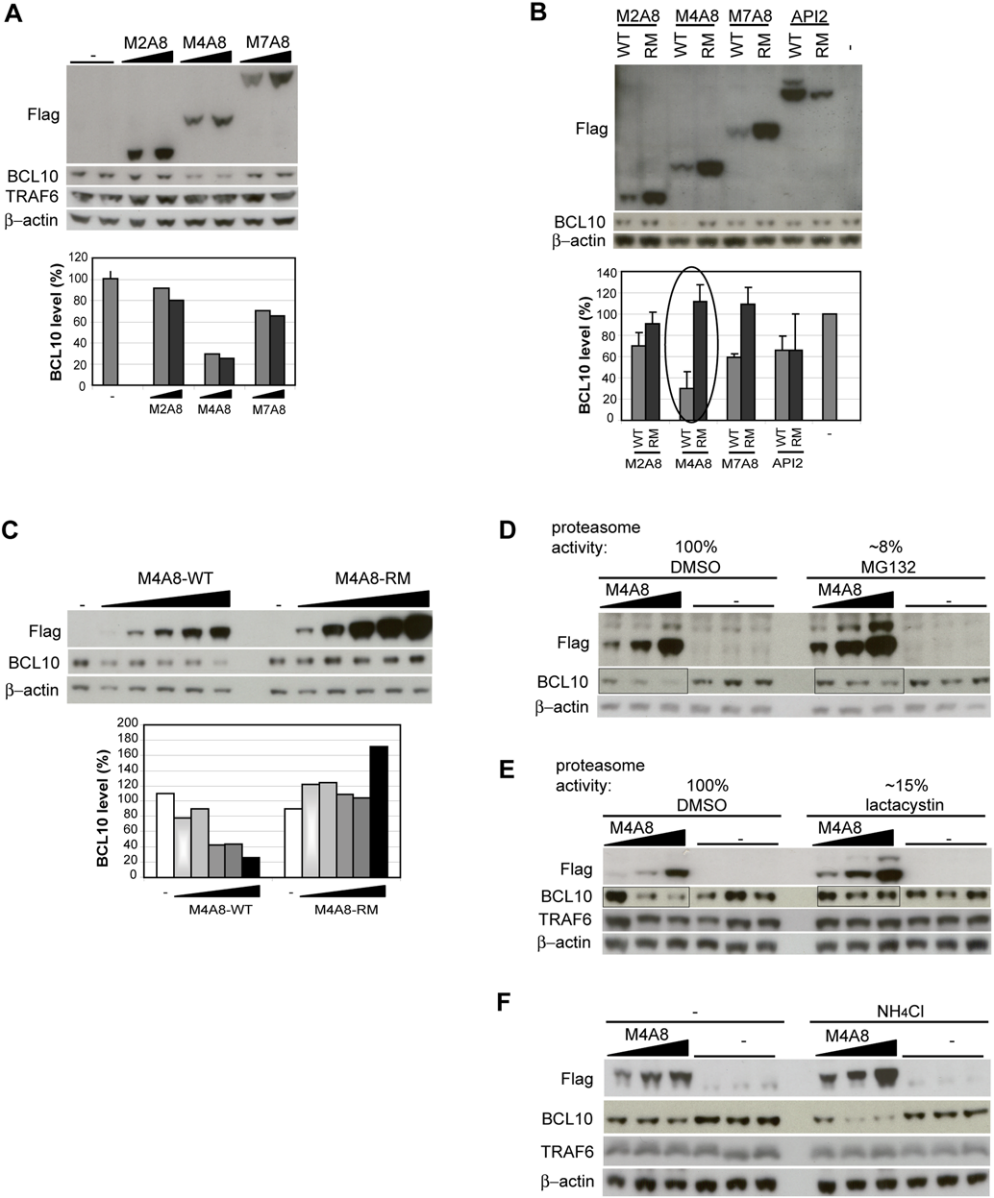
M4A8 induces BCL10 degradation by the proteasome. A, M4A8, and to a lesser extent M7A8, induces BCL10 degradation. Cell lysates of HEK-293T cells, transiently transfected with empty vector or increasing concentrations of Flag-tagged M2A8, M4A8 and M7A8 were immunodetected with α-Flag, α-BCL10, α-TRAF6 and α-β-actin. The BCL10 protein level (%) is compared to empty vector-transfected cells after normalization to β-actin. B, M4A8 requires a functional RING domain to induce BCL10 degradation. HEK-293T cells, transiently transfected with empty vector or Flag-tagged M2A8, M4A8, M7A8 and API2 (WT or RM) were lysed and immunodetected with α-Flag, α-BCL10 and α-β-actin. The BCL10 protein level (%) is compared to empty vector-transfected cells after normalization to β-actin and represents the mean of three independent experiments, with indicated standard deviation. C, HEK-293T cells were transiently transfected with empty vector or increasing concentrations of Flag-tagged WT or RM M4A8. Cell lysates were analyzed with α-Flag, α-BCL10 and α-β-actin. The BCL10 protein level (%) is compared to empty vector-transfected cells after normalization to β-actin. D–E, Proteasome inhibition reduces the degradation of BCL10 and of M4A8 itself. HEK-293T cells, transiently transfected with empty vector or increasing concentrations of Flag-tagged M4A8 were pretreated with DMSO, MG132 (D) or lactacystin (E) prior to harvesting. Cell lysates were analyzed with α-Flag, α-BCL10 and α-β-actin. F, Degradation of BCL10 or MALT1-API2 does not require the lysosome. HEK-293T cells, transiently transfected with empty vector or increasing concentrations of Flag-tagged M4A8 were left untreated or pretreated with NH_4_Cl prior to harvesting. Cell lysates were analyzed with α-Flag, α-BCL10, α-TRAF6 and α-β-actin.

The RING domains of IAPs are involved in ubiquitination-mediated protein degradation [10], [29], [39]–[42]. As we consistently observed a lower expression level for the wild-type MALT1-API2 proteins compared to their RING mutants (Fig 4B) even though more DNA was used for transfection (see Materials and Methods), we expected that also MALT1-API2 targets proteins for degradation. As shown in Fig 4A, expression of M4A8 in HEK-293T indeed reduced the BCL10 protein level. A smaller effect was seen for M7A8, while M2A8 did not affect the BCL10 level. Like its polyubiquitination, also the reduction of BCL10 levels by M4A8 and M7A8 was dependent on a functional RING domain, as the RING mutant (RM) had no effect (Fig 4BC). In contrast to M4A8 and M7A8, expression of wild-type API2 did not affect BCL10 protein levels, like its RING mutant (Fig 4B), which is in agreement with our observation that BCL10 does not interact with API2 in HEK-293T (Fig 3A). Similarly, none of the MALT1-API2 fusion variants had an effect on the TRAF6 protein level (Fig 4A), conform the absence of TRAF6 in MALT1-API2 protein complexes (Fig 2A).

To determine whether BCL10 degradation occurred via the proteasome or the lysosome, we tested the effect of inhibitors on BCL10 protein levels in HEK-293T cells expressing M4A8. Pretreatment of HEK-293T with MG132 prevented BCL10 degradation, even though the M4A8 level increased at the same time (Fig 4D). The same result was obtained when cells were pretreated with the more specific proteasomal inhibitor lactacystin (Fig 4E), while BCL10 degradation upon M4A8 expression could not be blocked with NH_4_Cl, an inhibitor of the lysosome (Fig 4F).

Altogether, these data indicate that the RING domain of M4A8 induces proteasome-mediated degradation of BCL10.

### Expression of *MALT1-API2* does not affect the BCL10 protein level and nuclear localization in t(11;18)(q21;q21)-positive *MALT* lymphoma

**Figure 5:**
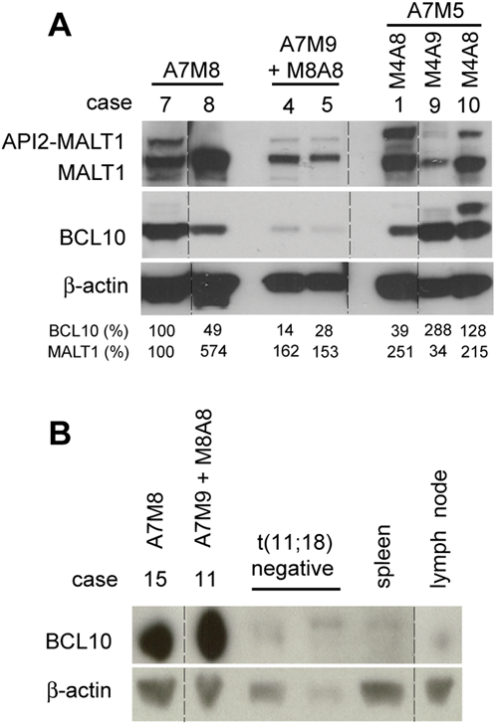
Expression of *MALT1-API2* does not affect the BCL10 protein level in t(11;18)(q21;q21)-positive MALT lymphoma. A, Lysates of 7 MALT lymphoma samples were immunodetected with α-MALT1-C, α-BCL10 and α-β-actin to evaluate the BCL10 protein level. Specifications about *API2-MALT1* and *MALT1-API2* transcript variants (from Table 1) are indicated. The BCL10 and MALT1 protein level (%) is compared to case 7 after normalization to β-actin. Different parts of the same blot were combined as indicated via the dotted lines. B, Immunoblot with α-BCL10 and α-β-actin on lysates from 4 MALT lymphoma cases (with specifications as indicated) and control spleen and lymph node samples. Different parts of the same blot were combined as indicated via the dotted lines.

To assess whether expression of *MALT1-API2* also affects BCL10 protein levels *in vivo*, we performed western blot analysis to evaluate BCL10 protein levels in t(11;18)(q21;q21)-positive MALT lymphoma samples with or without *MALT1-API2* expression. Expression of *M8A8* (case 4 and 5) was associated with reduced BCL10 levels when compared to two cases without the reciprocal *MALT1-API2* (case 7 and 8, Fig 5A). Analysis of an additional case however showed no effect of *M8A8* expression (case 11), although BCL10 expression was strongly upregulated compared to t(11;18)(q21;q21)-negative MALT lymphoma cases, control spleen or lymph node (Fig 5B). In contrast, two of the three samples with the *M4A8/9* variant, which induced the highest levels of BCL10 ubiquitination and degradation in HEK-293T cells, showed an increase in BCL10 expression levels (case 9 and 10), whereas a small reduction was observed in the remaining case (case 1, Fig 5A). Furthermore, immunohistochemistry showed a nuclear localization of BCL10 for all cases examined, with no differences between cases without (case 2, 7 and 8) or with expression of *M4A8/9* (case 1,6, 9 and 10) or *M8A8* (case 4 and 5) (Table 1; data not shown).

In conclusion, these data do not suggest an effect of *MALT1-API2* expression on BCL10 protein levels and nuclear staining in these MALT lymphomas.

### *MALT1-API2* reduces NF-κB activation by *API2-MALT1*

**Figure 6:**
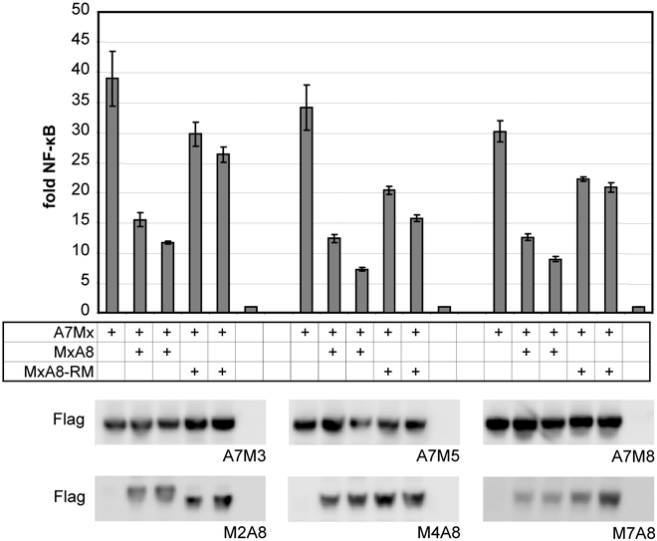
MALT1-API2 reduces NF-κB activation by API2-MALT1. NF-κB-dependent luciferase assay after co-expression of API2-MALT1 (A7M3/A7M5/A7M8) with its corresponding MALT1-API2 fusion variant (M2A8/M4A8/M7A8) in HEK-293T. NF-κB-dependent luciferase activity is represented as fold induction of vector-transfected cells. Shown is one representative experiment of 2, performed in triplicate, with mean and standard deviation. Lysates were immunodetected with α-Flag to confirm comparable API2-MALT1 expression in all cases.

Finally, the effect of MALT1-API2 expression on API2-MALT1-mediated NF-κB activation was examined by co-expression of the different API2-MALT1 fusion variants A7M3/A7M5/A7M8 in combination with their corresponding WT or RM MALT1-API2 variant M2A8/M4A8/M7A8 in HEK-293T. As shown in Fig 6, all MALT1-API2 fusion variants reduced NF-κB activation by API2-MALT1 to a similar extent. Although this was partly associated with an intact RING domain in MALT1-API2, MALT1-API2 also significantly affected NF-κB signalling independently of its E3 ubiquitin ligase activity.

## Discussion

The translocation t(11;18)(q21;q21) is a frequent genomic aberration associated with B-cell MALT lymphoma. Nowadays, it is generally accepted that the API2-MALT1 fusion protein expressed from the der(11) contributes to B-cell transformation and progression of MALT lymphomas via constitutive NF-κB activation. In contrast, not much is known about the reciprocal *MALT1-API2* fusion generated on the der(18) chromosome. Although it was initially reported that *MALT1-API2* is not consistently transcribed, we show here that the majority of MALT lymphomas with t(11;18)(q21;q21) do express in-frame *MALT1-API2* transcripts. Our data furthermore show that the RING domain of API2 provides MALT1-API2 with an E3 ubiquitin ligase activity. In addition, the inclusion of at least one Ig-like domain of MALT1 in MALT1-API2 results in the binding and ubiquitination of BCL10, which triggers its degradation by the proteasome *in vitro*. However, no correlation was found between the presence of a *MALT1-API2* transcript and the BCL10 expression level and nuclear staining in MALT lymphoma samples, most likely due to the efficient downregulation of MALT1-API2 protein levels via auto-ubiquitination.

Initial reports could not detect expression of *MALT1-API2* in the t(11;18)(q21;q21)-positive MALT lymphoma cases examined [32]–[34]. It is possible that the t(11;18)(q21;q21) is accompanied by deletions or other genomic aberrations that obstruct the generation of a *MALT1-API2* fusion on der(18). One group indeed reported deletions 5′ of the breakpoint on der(18) in all three MALT lymphoma cases examined [32]. Similarly, while only small intronic deletions were detected in the *API2* and/or the *MALT1* gene in three t(11;18)(q21;q21)-positive MALT lymphoma cases with expression of *MALT1-API2*, the failure to amplify a *MALT1-API2* transcript in two other cases correlated with a deletion of a substantial part of 3′-*API2* on der(18) ([35] and Baens, unpublished results). Such deletion involving the N-terminal part of the *MALT1* gene or the C-terminal part of the *API2* gene is however not a general observation in MALT lymphomas with t(11;18)(q21;q21), as a more recent genomic characterization of 19 t(11;18)(q21;q21)-positive MALT lymphomas demonstrated in 13 cases the generation of a genomic *MALT1-API2* fusion, with only small and mostly intronic deletions, duplications and/or insertions. Only in the 6 cases for which the amplification failed, deletions involving the entire C-terminal *API2* and/or the entire N-terminal *MALT1* gene were expected [36]. To address this apparent discrepancy between the presence of *MALT1-API2* fusions at the genomic level and the absence of *MALT1-API2* transcripts, we analyzed in this study *MALT1-API2* expression in 14 t(11;18)(q21;q21)-positive MALT lymphoma cases, of which 9 cases were previously reported to contain a *MALT1-API2* fusion at the genomic level. Surprisingly, RT-PCR and sequencing confirmed the expression of *MALT1-API2* in 7 out of these 9 cases (Table 1), which clearly shows that transcriptional silencing of *MALT1-API2* is not a general phenomenon in t(11;18)(q21;q21)-positive MALT lymphomas.

Based on the breakpoints in the *API2* and *MALT1* gene, a number of API2-MALT1 fusion variants have been described [7]. For all these variants, the anticipated reciprocal MALT1-API2 fusion protein always contains the N-terminal DD of MALT1 and the C-terminal RING domain of API2. The majority of cases however could contain in addition one or two Ig-like domains of MALT1 (Fig 1A, the M4A8 and M7A8/M8A8 fusion variants), as also demonstrated via genomic characterization of the der(18) chromosome [35], [36]. Here we show that the inclusion of at least one Ig-like domain mediates the binding of MALT1-API2 to BCL10, conform our previous observation that the DD and the Ig-like domains of MALT1 cooperate for BCL10 binding [26]. In contrast, TRAF6 did not interact with any of the fusion variants, despite the presence of the TRAF6 binding site associated with the second Ig-like domain (T6-Ig) of MALT1 in M7A8 and M8A8. This probably reflects conformational constraints imposed by the C-terminal domains of API2, as full-length MALT1 efficiently interacted with endogenous TRAF6 [26], or indicates the necessity of C-terminal sequences of MALT1 for efficient binding of TRAF6 to this site. In favour of the latter is our observation that deletion of the C-terminus of MALT1 in the API2-MALT1 fusion variant A7M3 also abolished TRAF6 binding to the T6-Ig site [26].

RING domains are able to catalyze the transfer of ubiquitin to a substrate. Our data show that the RING domain of API2 also enables MALT1-API2 to function as an E3 ubiquitin ligase capable of auto-ubiquitination, similarly as API2. Indeed, API2 is a known E3 protein involved in the ubiquitination-induced degradation of itself [9], [39] and of interacting proteins like RIP1 [40], TRAF1 [41] and SMAC [10]. Also BCL10 has recently been reported to be a target of API2-induced ubiquitination and degradation via binding to the BIR1 domain of API2 [29]. In this study, we were however unable to detect an interaction between API2 and endogenous BCL10, nor did we observe a RING-dependent effect of API2 overexpression on BCL10 stability. Although this confirms our previous observation that endogenous BCL10 does not interact with the BIR domains of API2 in API2-MALT1 [26], it needs to be determined whether the use of different cell types underlies this discrepancy or whether the interaction of BCL10 with API2 is stimulus-dependent [29], [43]. Nevertheless, we did detect a RING domain-dependent ubiquitination of BCL10 by M4A8, resulting in the degradation of BCL10 by the proteasome. Furthermore, while the inclusion of the second Ig-like domain of MALT1 in M7A8 somewhat increased its capacity to bind BCL10, this fusion variant could only modestly induce BCL10 ubiquitination and degradation, again suggesting conformational constraints.

Given the importance of NF-κB activation to the oncogenic behaviour of API2-MALT1 [27], the effect of MALT1-API2 expression on API2-MALT1-mediated NF-κB activation was examined *in vitro*. Interestingly, all MALT1-API2 fusion variants were found to severely reduce NF-κB activation by API2-MALT1, partly via the presence of an intact RING domain. This can however not be explained by the RING-mediated ubiquitination and degradation of BCL10, as BCL10 is not required for NF-κB activation by API2-MALT1 [26]. Further studies are thus needed to investigate whether the RING E3 ubiquitin ligase activity of MALT1-API2 affects specific proteins involved in API2-MALT1-mediated signalling to NF-κB. Furthermore, these studies could provide insight into the E3 ubiquitin ligase-independent mechanism of MALT1-API2 to reduce NF-κB activation by API2-MALT1. As this could possibly involve the DD of MALT1 and/or the CARD (caspase recruitment domain) of API2, the identification of interaction partners for these domains would form the subject of interesting future research. Moreover, it remains to be investigated whether expression of MALT1-API2 also reduces API2-MALT1-mediated NF-κB activation in t(11;18)(q21;q21)-positive MALT lymphomas, for example by analyzing its effect on the Lys63-linked polyubiquitination of IKKγ [23]. Unfortunately we were unable to do so in this study due to insufficient MALT lymphoma material available.

In addition to NF-κB activation by API2-MALT1, also the increased BCL10 protein level [29] and the unusual nuclear localization of BCL10 [30], [31] have been suggested to contribute to the oncogenic character of the t(11;18)(q21;q21) in MALT lymphomas. The destabilizing effect of MALT1-API2 on BCL10 *in vitro* thus suggests that MALT1-API2 could also interfere in this way with the development of t(11;18)(q21;q21)-positive MALT lymphomas. However, the expression of *MALT1-API2* did not affect the nuclear staining of BCL10 in our MALT lymphoma cases analyzed. In addition, we could not confirm decreased levels of BCL10 in MALT lymphoma B-cells expressing *M4A8*, this in sharp contrast to our *in vitro* observations. As MALT1-API2 is very unstable being an efficient target of its own E3 ubiquitin ligase activity, this suggests that its protein level in MALT lymphoma might be too low to affect BCL10 stability and localization. Unfortunately, we were not able to validate this further due to the lack of good quality antibodies able to detect the MALT1-API2 protein.

In conclusion, our data show that the MALT1-API2 fusions with one or both Ig-like domains of MALT1 can bind BCL10 and induce its ubiquitination and proteasome-mediated degradation via the RING domain of API2. However, MALT1-API2 is also an efficient target of its own E3 ubiquitin ligase activity. This property of MALT1-API2 most likely limits its protein level in t(11;18)(q21;q21)-positive B-cells and makes a possible effect on MALT lymphoma development via destabilization of BCL10 unlikely.

## Materials and Methods

### Constructs

Vectors enabling expression of biotinylated proteins (pcD-F-bio; pcD-HA-bio) were constructed as described [26] and used to generate constructs for MALT1, BCL10, API2, ubiquitin (Ub), the MALT1-API2 fusion variants M2A8, M4A8, M7A8 and M8A8 (fusion of respectively exon 2, 4, 7 and 8 of *MALT1* with exon 8 of *API2*, Fig 1A) and their RING mutants (RM). PCR-based mutagenesis was used to introduce the triple mutation of the Zinc coordinating Histidine and the flanking Cysteines (C572S/H574A/C578S) in the RING domain of API2, which was subcloned in API2 and the MALT1-API2 constructs to create the corresponding RM.

### Cell culture, transfection and NF-κB reporter assays

Monoclonal SSK41 B-cell lines stably expressing Flag-tagged BirA, alone or in combination with a Flag-bio-tagged M4A8, were generated and cultured as described [26]. HEK-293T cells were cultured and transfected as described [26]. For transfections involving MALT1-API2, a double amount of DNA of the wild-type MALT1-API2 variants compared to their RING mutants was used to obtain more equal expression levels. After 24 hours, cells were harvested in 1×PBS and lysed in a non-denaturing NDLB lysis buffer, as described [26]. NF-κB reporter assays were performed as described [26].

### Inhibition of the lysosome and the proteasome

To inhibit the proteasome, cells were treated for 4 to 7 hours with 50 µM MG132 (VWR International) or with 25 µM lactacystin (Cayman, Bio-Connect, Huissen, The Netherlands) prior to harvesting. DMSO treatment was included as a control. To measure proteasome activity, 100 µg of cell lysate (20 mM Tris-Cl pH 8.0, 0.5 mM EDTA, 0.035% SDS) was incubated with 200 µM Suc-LLVY-AFC (Sigma-Aldrich, Bornem, Belgium) for 30 min on 37°C, after which released AFC was quantified (excitation 390 nm, emission 460 nm) using a FLUOstar Galaxy Plate Reader (BMG Labtechnologies GmbH, Offenburg, Germany). To inhibit the lysosome, cells were treated for 4 hours with 50 mM NH_4_Cl prior to harvesting.

### Bio-IP and phosphatase treatment

The biotin-immunoprecipitation (bio-IP) method [38] was performed as described [26]. Where indicated, cell lysates were denatured in the presence of 1% SDS prior to bio-IP. To remove phosphorylations following bio-IP, where indicated, beads were resuspended in 1× lambda protein phosphatase (λ-PPase) reaction buffer and treated with λ-PPase (New England Biolabs, Westburg BV, Leusden, The Netherlands).

### SDS-PAGE and western blot analysis

SDS-PAGE and western blot analysis were performed as described [26]. Antibodies used for immunodetection of the Flag epitope (M2) and of β-actin (clone AC-15) were from Sigma-Aldrich, of the HA epitope (12CA5) from Roche Diagnostics Belgium (Vilvoorde, Belgium) and of BCL10 (sc-5273), TRAF6 (sc-7221) and Ub (sc-8017) from Santa Cruz Biotechnology (Tebu-bio, Boechout, Belgium). Anti-MALT1-C is a rabbit polyclonal antiserum raised against amino acids 731–824 of MALT1 [24]. The signal was visualized with the enhanced chemoluminescence (ECL) system (Perkin Elmer, Zaventem, Belgium) and, where indicated, quantified using the Advanced Image Data Analyzer (AIDA) Software (Raytest BeNeLux B.V., Tilburg, The Netherlands). Where indicated, membranes were stripped (62.5 mM Tris-Cl pH 6.7, 2% SDS and 100 mM β-mercaptoethanol) for 30 min at 60°C and, prior to reprobing, analyzed via western blot detection with the appropriate secondary antibody to verify complete stripping.

### MALT lymphoma samples

Fifteen cases of t(11;18)(q21;q21)-positive MALT lymphoma with fresh-frozen tumour tissues were collected, of which 10 (case 1 to 10) from the Department of Pathology, University of Cambridge, UK and 5 (cases 11 to 15) from the Department of Morphology and Molecular Pathology, University of Leuven, Belgium (Table 1). t(11;18)(q21;q21) and the *API2-MALT1* fusion junction at the transcript level were identified previously (Table 1; [35], [36]; unpublished for cases 2, 4, 13–15). The breakpoint on the der(18) was identified previously for 9 cases (Table 1; [35], [36]). Two t(11;18)(q21;q21)-negative MALT lymphoma cases and control spleen and lymph node samples were retrieved from the Department of Morphology and Molecular Pathology, University of Leuven, Belgium. For western blot analysis, tumour tissues were lysed in 100 mM Tris-Cl pH 8.0, 300 mM NaCl, 0.04% NaN_3_, 0.2% SDS, 2% NP-40, 1% Na-deoxycholate, 2 mM EDTA, 100 mg/ml PMSF and 1 mg/ml leupeptin. RNA isolation and cDNA synthesis were performed as described ([30] for cases 1–10 and [35] for cases 11–14). BCL10 immunohistochemistry was performed as described [30].

### (Quantitative) RT-PCR and sequence analysis of MALT1-API2 transcripts

To determine in our collection of t(11;18)(q21;q21)-positive MALT lymphoma samples the *MALT1-API2* fusion variant at transcript level, two different RT-PCR reactions were performed: one with primers MALT1-F1 (in *MALT1* exon 2) and API2-R1 (in RING domain of *API2* exon 10), followed by a second round of nested PCR with primers MALT1-F2 (in *MALT1* exon 2) and API2-R2 (in RING domain of *API2* exon 10) (Fig 1B), and a second one with primers MALT1-F3 (in *MALT1* exon 4) and API2-R1, followed by a nested PCR with primers MALT1-F4 (in *MALT1* exon 4) and API2-R2 (Fig S1AB). RT-PCR for *MALT1* (with MALT1-F5 and MALT1-R1) and *GAPDH* (with GAPDH-F and GAPDH-R) were performed to check for RNA quality. The PCR products in Fig 1B were sequenced using primers MALT1-F2 and API2-R2. Big Dye Terminator Cycle Sequencing Reactions were analyzed on an ABI PRISM 3130 Genetic Analyzer (Applied Biosystems, Lennik, Belgium). Quantitative RT-PCR was performed with the LightCycler 480 SYBR Green I master mix (Roche Diagnostics Belgium) and analyzed using the comparative dCt method to check expression levels of *HPRT1* (HPRT1-F/R), *M8A8* (M8A8-F/R) and *A7M9* (A7M9-F/R) for case 11 and *M4A8* (M4A8-F/R) and *A7M5* (A7M5-F/R) for case 12. Primer sequences are shown in Table 2.

**Table 2:** Sequence of the primers used.

Primer	Sequence	Gene
MALT1-F1	5′-TTGCCTAGACCTGGAGCAGT	*MALT1*
MALT1-F2	5′-GATTTCCTGCAGGCTATGGA	*MALT1*
MALT1-F3	5′-GCAGGCTTTTATGTCTGTCG	*MALT1*
MALT1-F4	5′-TTGAATTCAGCCAGTGGTCA	*MALT1*
MALT1-F5	5′-GTGGATTTGGAACACCAAGG	*MALT1*
MALT1-R1	5′-CCACTGTAGATTCCGCACAA	*MALT1*
API2-R1	5′-GAAGGAGCACAATCTTTGCAT	*API2*
API2-R2	5′-GGACACTTCTTTGTCCATACACAC	*API2*
GAPDH-F	5′-AATCCCATCACCATCTTCCA	*GAPDH*
GAPDH-R	5′-ACAGTCTTCTGGGTGGCAGT	*GAPDH*
HPRT1-F	5′-TGACACTGGCAAAACAATGCA	*HPRT1*
HPRT1-R	5′-GGTCCTTTTCACCAGCAAGCT	*HPRT1*
M8A8-F	5′-GCACTGAAGATGAATTAAATAATCTTGG	*MALT1*
M8A8-R	5′-TGGAATTACACAAGTCAAATGTTGAAA	*API2*
A7M9-F	5′-AGAAGATGAAATAAGGGAAGAGGAG	*MALT1*
A7M9-R	5′-TTCGCCAAAGGCTGGTC	*API2*
M4A8-F	5′-TCAGCCAGTGGTCACAG	*MALT1*
M4A8-R	5′-GCCATTCTATTCTTCCGGATTAATAATAAA	*API2*
A7M5-F	5′-GGAAGAGGAGAGAGAAAGAGC	*MALT1*
A7M5-R	5′-CAACTTGGATTCAGAGACGC	*API2*

## Supporting Information

Figure S1 RT-PCR analysis of *MALT1-API2* expression in t(11;18)(q21;q21)-positive MALT lymphoma. A, Shown are the amplification products of RT-PCR reactions with primers MALT1-F3 and API2-R1 on cDNA extracted from 14 t(11;18)(q21;q21)-positive MALT lymphoma cases (see Table 1). A PCR on pcD-F-M4A8 and pcD-F-M8A8 was performed as positive control. B, A second round of nested PCR was performed with primers MALT1-F4 and API2-R2. For primer sequences, see Table 2.
(1.63 MB PDF)
