# Supplementary figures and images for: Auto-Ubiquitination-Induced Degradation of MALT1-API2 Prevents BCL10 Destabilization in t(11;18)(q21;q21)-Positive MALT Lymphoma
Source: PLoS One. 2009 Mar 12;4(3):e4822. doi: 10.1371/journal.pone.0004822 (PMC2652110; doi:10.1371/journal.pone.0004822)

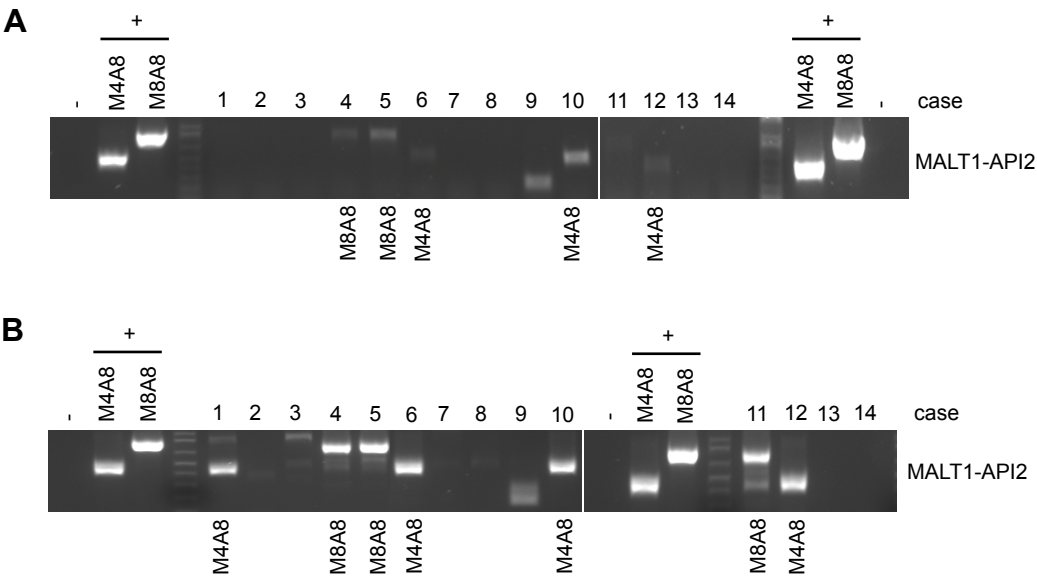

Supplement: Figure S1 — RT-PCR analysis of MALT1-API2 expression in t(11;18)(q21;q21)-positive MALT lymphoma. A, Shown are the amplification products of RT-PCR reactions with primers MALT1-F3 and API2-R1 on cDNA extracted from 14 t(11;18)(q21;q21)-positive MALT lymphoma cases (see Table 1). A PCR on pcD-F-M4A8 and pcD-F-M8A8 was performed as positive control. B, A second round of nested PCR was performed with primers MALT1-F4 and API2-R2. For primer sequences, see Table 2. (1.63 MB PDF) [file pone.0004822.s001.pdf]
